# BSR and Full-Length Transcriptome Approaches Identified Candidate Genes for High Seed Ratio in *Camellia vietnamensis*

**DOI:** 10.3390/cimb45010022

**Published:** 2022-12-31

**Authors:** Bing-Qing Hao, Hong-Ze Liao, Ying-Ying Xia, Dong-Xue Wang, Hang Ye

**Affiliations:** 1 Research Institute of Tropical Forestry, Chinese Academy of Forestry, 682 Guangshan Road, Guangzhou 510630, China; 2 Guangxi Forestry Research Institute, 23 Yongwu Road, Nanning 530002, China; 3 Guangxi Key Laboratory of Special Non-Wood Forest Cultivation & Utilization, Improved Variety and Cultivation Engineering Research Center of Oil-Tea Camellia in Guangxi, Nanning 530002, China; 4 School of Marine Sciences and Biotechnology, Guangxi Minzu University, 158 West Daxue Road, Nanning 530008, China

**Keywords:** *Camellia vietnamensis*, high seed ratio, bulked segregant RNA analysis, full-lengthtranscriptome sequencing

## Abstract

(1) Background: *C. vietnamensis* is very suitable for growth in the low hilly areas of southern subtropical regions. Under appropriate conditions, the oil yield of *C. vietnamensis* can reach 1125 kg/ha (the existing varieties can reach 750 kg/ha). Moreover, the fruit of *C. vietnamensis* is large and the pericarp is thick (>5 cm). Therefore, a high seed ratio has become the main target economic trait for the breeding of *C. vietnamensis.* (2) Methods: A half-sibling population of *C. vietnamensis* plants with a combination of high and low seed ratios was constructed by crossing a *C. vietnamensis* female parent. Bulked segregant RNA analysis and full-length transcriptome sequencing were performed to determine the molecular mechanisms underlying a high seed ratio. (3) Results: Seed ratio is a complex quantitative trait with a normal distribution, which is significantly associated with four other traits of fruit (seed weight, seed number, fruit diameter, and pericarp thickness). Two candidate regions related to high seed ratio (HSR) were predicted. One spanned 140.8–148.4 Mb of chromosome 2 and was associated with 97 seed-yield-related candidate genes ranging in length from 278 to 16,628 bp. The other spanned 35.3–37.3 Mb on chromosome 15 and was associated with 38 genes ranging in length from 221 to 16,928 bp. Using the full-length transcript as a template, a total of 115 candidate transcripts were obtained, and 78 transcripts were predicted to be functionally annotated. The DEGs from two set pairs of cDNA sequencing bulks were enriched to cytochrome p450 CYP76F14 (KOG0156; GO:0055114, *HSR4*, *HSR7*), the gibberellin phytohormone pathway (GO:0016787, *HSR5*), the calcium signaling pathway (GO:0005509, *HSR6*), the polyubiquitin-PPAR signaling pathway (GO:0005515, *HSR2*, *HSR3*), and several main transcription factors (bZIP transcription factor, *HSR1*) in *C. vietnamensis*.

## 1. Introduction

*Camellia vietnamensis* T.C.Huang (Theaceae) is an evergreen woody shrub commonly found and cultivated in northern Vietnam and southern China [1]. And oil tea species, olive tree (*Olea europaea*), oil palm (*Elaeis guineensis*), and coconut palm (*Cocos nucifera*) are the world’s four largest woody oil plants [2,3]. Camellia oil is obtained from fruit seeds and has high nutritional, healthcare, and economic value [4,5]. Camellia oil has a fatty acid content of up to 90% and an oleic acid content over 80%, and is rich in squalene, vitamin E, sterols, and polyphenols [6,7,8]. The *C. vietnamensis* fruit is relatively large, with an average mass of 38 g and maximum mass of 300 g, but its peel is thick, and the average fresh seed ratio is less than 30%. Therefore, from an economic perspective, a high seed ratio has become the main target for breeding of *C. vietnamensis* varieties. The seed ratio of a *C. vietnamensis* population varies significantly, ranging from 24.6% to 34.3% in 90% of plants and being over 40% in less than 0.8% of plants. Quantitative traits are genetically controlled by many genes, each of which has a relatively small effect on the phenotype, but are largely influenced by the environment [9]. Conventional breeding methods select for and improve traits based on the plant’s phenotype. Molecular markers were developed to assess the genome, and identify genetic regions that are implicated in traits due to the genes located within them. Bulked segregant RNA (BSR) analysis is a form of cluster segregation based on second-generation, high-throughput sequencing technology, which allows for gene targeting in F2-generation populations and is not limited by population size, provided there are enough extreme traits in the population to allow for the cost-effective primary targeting of genes [10,11].

Guangxi is one of the three major areas of camellia oil production in China, with a planting area of 570,000 hectares. In recent years, it has been found that camellia oil faces significant difficulties, such as a low afforestation survival rate and slow early growth in the high-temperature and high-humidity areas south of the Tropic of Cancer, and the existing superior varieties cannot meet the development needs of the camellia oil industry in Guangxi. However, the investigation found that *C. vietnamensis* is very suitable for growth in the low hilly areas of the southern subtropics. Under appropriate conditions, the oil yield of *C. vietnamensis* can reach 1125 kg/ha (the existing varieties can reach 750 kg/ha). Moreover, the fruit yield of an excellent individual strain can reach as high as 5.73 kg/m^2^, and the oil yield can reach 1375.2 kg/hm^2^ according to a 60% effective area, which indicates the high potential of the oil plant. The fruit of *C. vietnamensis* is large, with an average single fruit weighing 38.0 (25~140) g, with a maximum of 300 g; the pericarp is thick (>5 cm); and the average fresh seed ratio is less than 30%. Therefore, “high seed ratio” has become the main target economic trait for the breeding of *C. vietnamensis.* Since 2005, this project team has completed phenotypic diversity surveys (flower, fruit, and leaf) in 13 plots for *C. vietnamensis,* including Vietnam, Guangdong, Hainan, and Guangxi. The survey data show that the fresh seed ratio of fruits in the seedling population of *C. vietnamensis* was significantly different. Plants with a fresh seed ratio of 24.6–34.3% constituted more than 90%, while plants with a fresh seed ratio of >40% represented less than 0.8% (unpublished data).

Livaja et al. (2013) [12] combined high-throughput sequencing technology with BSR and de novo assembly technology for the first time to successfully perform efficient marker enrichment and fine-mapping in specific genomic regions of a non-model sunflower, and they conducted an anti-bacterial assay via single-nucleotide polymorphism (SNP) validation to screen downy mildew candidate genes. Since then, research on the association genetics of high-value quantitative traits based on candidate genes in crops has progressed rapidly. For example, Haixia Tang et al. (2015) [13] combined mRNA sequencing (RNA-Seq) with bulked segregant analysis (BSA) to screen 199 genes related to high growth in high-growth (GD) and low-growth (BD) groups of Ginkgo biloba half-sibling families. By integrating BSA and RNA-seq results, a total of 29 candidate genes associated with the slow-melting flesh trait in peach located in the mapping site were discovered (Chen C et al., 2021) [14]. A total of 291 unigene sequences related to early and late flowering in tree peony were finally screened out by BSR-seq (bulked segregant RNA-seq) association analysis (Hou X et al., 2018) [15]. Tian-Yu Mao et al. (2020) identified 30 candidate genes related to weeping and upright progeny in an F1 population of Prunus mume [16]. Zheng identified six major functional genes and created two genetic variation networks for the root growth angle with RNA sequencing (RNA-seq) and parental resequencing in apple [17] (Zheng, C et al., 2020). Finally, through the joint analysis of network and protein–protein interaction, MdSAUR2, MdSAUR29, MdSAUR60, MdSAUR62, MdSAUR69, MdSAUR71, and MdSAUR84 were screened as the main candidate genes for regulating RGA (Zhou, Y. 2022) [18]. Additionally, 33 QTLs were identified for carbon isotope composition in apple under both well-watered and drought-stressed conditions (Wang, H., 2018) [19].

In this study, a half-sibling population of *C. vietnamensis* plants with a combination of high and low seed ratios is reported. Seed ratio is a complex quantitative trait. We found four traits (seed weight, seed number, fruit diameter, and pericarp thickness) that were highly significantly correlated with seed ratio. The conjunctive analyses of full-length transcriptome sequencing and BSR-Seq were performed with the F2 separation progeny to study the molecular mechanism of high seed ratio formation in *C. vietnamensis*. The results presented here can provide a foundation for uncovering the mechanisms responsible for the *C. vietnamensis* seed ratio trait to accelerate the breeding of new varieties with a higher seed ratio.

## 2. Materials and Methods

### 2.1. Plant Material

Of the 300 F1 half-sib progeny individuals, 20 plants with a high seed ratio (43.6% to 50.7%) and 20 individuals with a low seed ratio (15.86% to 27.18%) were selected to establish segregation bulks, which were planted at the Oil Tea Germplasm Repository of the Guangxi Forest Research Institution (22°55′51″ N, 108°20′03″ E).

Half-sib progeny individuals: The female parent plant of the 300 offspring is ‘honggu08’, and the male parent is not known, because in natural state, *C. vietnamensis* is heterogeneously pollinated due to its self-incompatibility, so the source of its pollen (parent) is unclear.

Sampling was carried out in early October, so that *C. vietnamensis* oil fruits were fully expanded. In total, 100 g of fruit was sampled from each designated plant in four directions, then immediately put into liquid nitrogen for storage. The fruits were mixed and ground to extract RNA from each plant. Additionally, the RNAs that met the requirements in terms of completeness and purity were mixed in equal amounts to construct high-seed-ratio bulk (pool 1) and low-seed-ratio bulk (pool 2). Then, RNAs from the fruit, flower, and leaf of the maternal plant were mixed in equal amounts for full-length transcriptome sequencing. All samples were flash frozen in liquid nitrogen and stored at −80 °C.

### 2.2. Trait Evaluation

We collected 30 fruits from each plant and measured the following variables: fruit mass, pericarp thickness, and seed mass. The average fresh seed ratio of the plant was calculated using the following formula:

$$Fresh\;seed\;ratio=\frac{Fresh\;seed\;weight}{Fruit\;weight}$$

### 2.3. Full-Length Transcriptome Sequencing

To compare characteristics based on the fresh seed ratio, full-length transcripts of the high- and low-seed-ratio samples were obtained via full-length transcriptome sequencing. This procedure used the standard protocol of PacBio sequel II, which is based on single-molecule real-time (SMRT) sequencing technology. We used the SMRT Link software to filter and process the raw sequencing output data (stored in the FASTQ format) and obtained full-length non-chimeric (FLNC) sequences by checking for the presence of a chimera, primer, and 3′ terminal poly A sequences. The splice, primer, and poly A were removed from the FLNC sequences. In addition, the FLNC sequences were corrected for accuracy, and the final high-quality, full-length transcripts were combined to obtain high-quality, non-redundant, full-length transcripts for subsequent analysis.

### 2.4. Prediction and Functional Annotation of Full-Length Transcripts and Their Open Reading Frames

The open reading frames (ORFs) of full-length transcripts were predicted using the TransDecoder software (https://transdecoder.github.io/) accessed on 14 March 2022 [20]. For transcripts with multiple ORFs, the longest ORF was selected for subsequent functional analysis. The functional annotation of full-length transcripts was based on the following seven databases: Swiss-Prot (UniProt Consortium) [21], Protein Family (Pfam) [22], the Kyoto Encyclopedia of Genes and Genomes (KEGG) [23], Gene Ontology (GO) [24], Non-Redundant (NR) [25], EuKaryotic Orthologous Group (KOG) [26], and TrEMBL [27]. The PLEK, CPC 2.0, and CPAT programs [28] were used to calculate the coding potential of sequences of the full-length transcripts that were longer than 200 nt. The intersection of the three results was then determined, excluding transcripts with conserved structural domain annotations in the Pfam database [29].

### 2.5. BSR-Seq Analysis

For BSR-seq analysis, three cDNA pools were constructed using the female parent bulk and the two F1 bulks (pool 1 and pool 2); total RNA was isolated from fresh leaves according to the standard protocol of Illumina HiSeq × 10 [30,31]. Raw sequencing data contained splice information, low-quality bases, and undetected bases (expressed as N) that could cause confusion in subsequent analysis. We thus used Fast QC and Trimmomatic software [32,33] to check and control the quality of the raw data by (1) filtering out the reads with splice sequences and (2) filtering out the reads and paired-end reads when the N content and the number of low-quality (≤5) bases in the single-end sequencing reads exceeded 10% and 50%, respectively.

### 2.6. Analysis of BSR-Seq Data

High-quality sequences were aligned and mapped to the reference *C. oleifera* var. genome (NCBI:ASM2231669v1) [34] using the HISAT2 program with default parameters [35]. High-quality sequences were aligned and mapped to a full-length transcriptome sequence of *C. vietnamensis* using Bowtie2 software [36]. The results were converted to fragments per kilobase of transcript per million fragments mapped (FPKM) [37] to obtain the transcript expression levels. The FPKM gene expression unit denotes the average number of read pairs per kilobase per million read pairs of a given transcript, which considers the effects of both sequencing depth and gene length on read counts and is the most used method for estimating gene expression levels.

### 2.7. SNP and InDel Detection

The Genome Analysis Toolkit (GATK) [38] was used to identify SNPs and small indels across parental lines and bulks [39]. On the basis of the localization of the sequenced reads on the reference sequence, local realignment was performed using GATK 4.0 and variant detection using GATK 4.0 and SAMtools [5]. To ensure accurate variant loci identification, the intersection of the variant loci obtained using the two detection methods was used to obtain the final set of variant loci. Variants with a sequencing depth greater than 4 in each sample and exhibiting polymorphism between the two mixed bulks were retained for subsequent analysis using the GATK. Before association analysis, the original SNPs and indel loci were filtered out based on the following criteria: SNPs with multiple genotypes, loci with a read support less than 10, loci with no polymorphism between bulks, and loci with deletions in a sample species.

### 2.8. Association Analysis

The Euclidean distance (ED)

$$\mathrm{ED}=\sqrt{{\left(\mathrm{Amut}-\mathrm{Awt}\right)}^{2}+{\left(\mathrm{Cmut}-\mathrm{Cwt}\right)}^{2}+{\left(\mathrm{Gmut}-\mathrm{Gwt}\right)}^{2}+{\left(\mathrm{Tmut}-\mathrm{Twt}\right)}^{2}}$$

This Algorithm is a method for assessing a trait’s region of association using significant difference markers between pools. Amut, Cmut, Gmut, and Tmut are the frequencies of A, C, G, and T bases, respectively, in the high-seed-ratio bulks, and Awt, Cwt, Gwt, and Twt are the frequencies of A, C, G, and T bases in the low-seed-ratio bulks. Theoretically, the two hybrid pools constructed via BSA tend to be identical, except for differences in the target trait-related loci, such that the ED value for non-target loci tends to be zero. A larger ED value indicates a greater difference between the two hybrid pools for that marker. To eliminate background noise, raw ED values were multiplied to the power of five and fitted using the mean ED value of the variant loci on each transcript.

### 2.9. Functional Annotation and Expression Analysis of Associated Genes or Transcripts

A total of seven databases (Swiss-Prot, Pfam, KEGG, GO, NR, KOG, and TrEMBL) were used for the functional annotation of associated genes or transcripts. The number of reads per sample compared with each gene was obtained using the results of matching the mixed pool data to the full-length transcripts or reference genome, and FPKM transformation was performed to obtain the expression levels of the transcripts.

## 3. Results

### 3.1. Phenotypic Data

**Figure 1:**
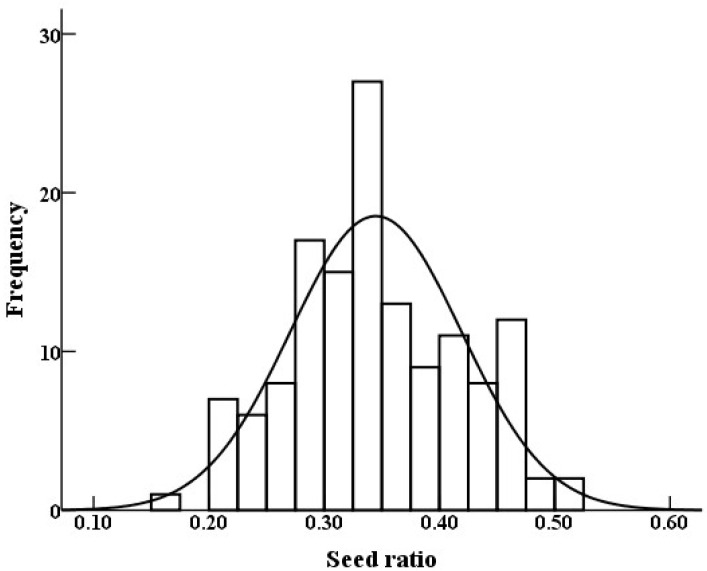
Normal distribution of seed ratio in *C. vietnamensis* population.

**Figure 2:**
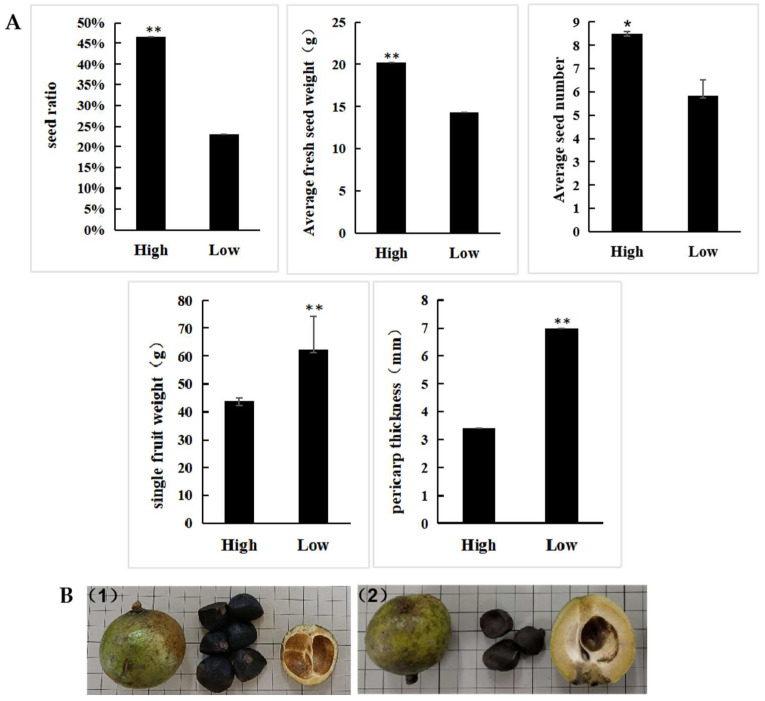
Phenotypic data (**A**) and architecture (**B**) of high- and low-seed-ratio *C. vietnamensis* fruits. (**1**) Architecture of high-seed-ratio fruit; (**2**) Architecture of low-seed-ratio fruit. Asterisks indicate significant differences: * *p* < 0.05 and ** *p* < 0.01.

The seed ratio of the 300 F1 half-sib progeny plants exhibited a normal distribution and ranged from 15.86% to 50.70%, with a mean value of 34% (Figure 1). The mean pericarp thickness and fruit weight of the high-seed-ratio bulks (3.4 cm and 43.67 g, respectively) were significantly lower than those of the low-seed-ratio bulks (6.9 cm and 62.45 g, respectively; *p* < 0.01). However, the seed numbers per fruit were significantly higher in the high-seed-ratio bulks than in the low-seed-ratio bulks (*p* = 0.012; Figure 2A). Morphologically, the fruits of the two mixed pools differed significantly, mainly in seed number and pericarp thickness, which is consistent with the analysis of phenotypic data (Figure 2B).

### 3.2. Transcriptomic Analysis via SMRT Sequencing

The total length of the obtained non-redundant full-length transcripts was 12,403,692 kb, spanning 14,152 sequences. These 14,152 sequences comprised 9835 transcripts shorter than 1 kb (69.5%), 3935 transcripts longer than 1 kb and shorter than 2 kb (27.8%), 350 transcripts longer than 2 kb and shorter than 3 kb (2.5%), and 32 transcripts longer than 3 kb (0.2%) (Table 1). The maximum transcript length was 4719 bp, the minimum was 107 bp, the average was 876 bp, the median was 770 bp, N50 was 997 bp, and N90 was 515 bp. The PLEK, CPC 2.0, and CPAT programs were used to determine the sequences of full-length transcripts longer than 200 nt. The results were intersected, excluding transcripts with conserved structural domains in the Pfam database, and a total of 2785 long-stranded, non-coding RNAs (lncRNAs) were obtained (Figure S1). A total of 17,519 simple sequence repeats (SSRs) were obtained using the misa.pl program, 65.32% of which comprised 11,444 microsatellite SSRs (6372 mononucleotide; 10,271 dinucleotide; 4718 trinucleotide; 235 tetranucleotide; 48 pentanucleotide; and 101 hexanucleotide repeats). A total of 12,637 ORFs were obtained and annotated for the GO function.

By comparing the seven databases (Swiss-Prot, Pfam, KEGG, GO, NR, KOG, and TrEMBL), 10,178 transcripts were functionally annotated. In total, 4430 transcripts were annotated in the GO; 10,099 in the TrEMBL; 4253 in the KEGG; 6060 in the KOG; 7372 in the Pfam; 8007 in the Swiss-Prot; and 10,091 in the NR database. The GO terms were divided into three ontological categories: molecular function, cellular component, and biological process. Specifically, the GO annotation results show that the cell part and protein-containing complex were enriched under the cellular component category, binding and catalytic activity were enriched under the molecular function category, and metabolic and cellular processes were enriched under the biological process category (Figure S2). The KEGG transcript annotation results were divided into five categories: cellular processes, environmental information processing, genetic information processing, metabolism, and organismal systems. The main transcript enrichment pathways were carbohydrate metabolism, signal transduction, and translation (Figure S3).

### 3.3. Differential Expression of Transcripts in Relation to Seed Ratio

The raw next-generation sequencing data from pool 1 and pool 2 were 9.3 Gb and 11.2 Gb, respectively, with a Q20 of 97% and 96% and Q30 of 93% and 92%, respectively, and a GC content ranging from 46% to 48%. The raw data from pools 1 and 2 were compared with the full-length transcripts using HISAT2 software, with matching rates of 60.14% and 62.06%, respectively, and an average sequencing depth of approximately 200× for all bases on the genome (Table 1). The SNP and InDel assays resulted in 154,265 loci (154,265 SNP and 18,352 indel loci) spanning 8872 transcripts.

**Figure 3:**
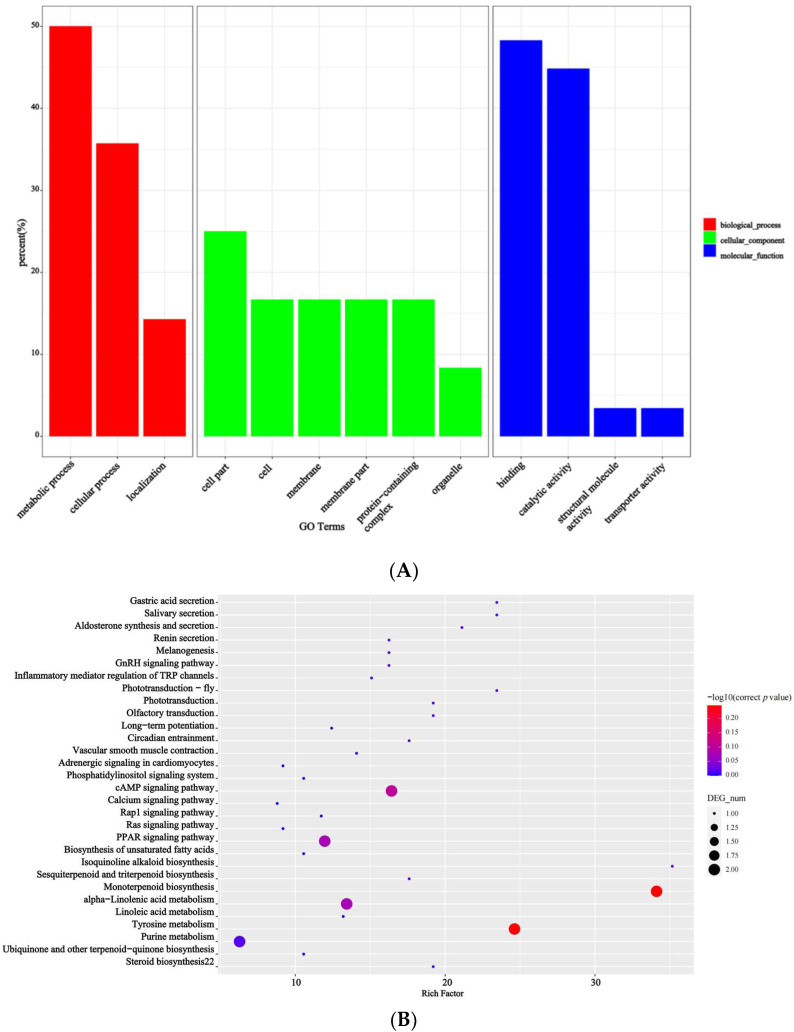
The enriched GO and KEGG terms of the differential expression of transcripts. (**A**) The enriched GO terms of the differential expression of transcripts. (**B**) The enriched KEGG terms of the differential expression of transcripts.

The association analysis of these loci yielded 115 candidate transcripts, of which 78 transcripts were predicted to be functionally annotated (based on the Swiss-Prot, Pfam, KEGG, GO, NR, KOG, and TrEMBL databases) according to the association threshold, that is, the mean of all fitted loci (median) +5 SD (Figure 3). The product of differently expressed transcripts (DEPs) was predicted to contain agglutinin, the proteolytic enzyme inhibitor/seed storage/lipid transfer protein family, photosystem I reaction center subunit IV, chlorophyllase enzyme, and Erg28 protein in the Pfam pathway (Figure 3).

### 3.4. Differential Expression of Genes in Relation to Seed Ratios

**Figure 4:**
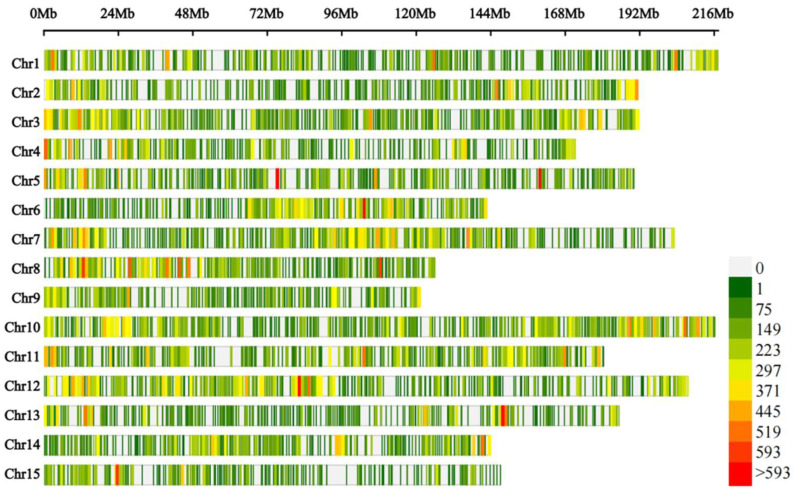
Distribution of variant loci in two seed ratio bulks of *C. vietnamensis* on *C. oleifera* var. chromosomes.

**Figure 5:**
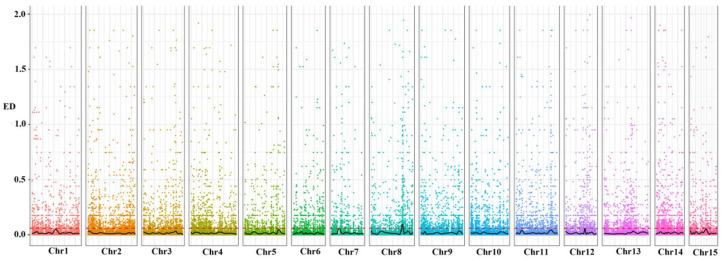
Distribution of ED association values of two bulks of *C. vietnamensis* on *C. oleifera* var. chromosomes. Note: the horizontal coordinates are the chromosome names, the different-colored dots represent the ED values of each SNP locus, the black line is the fitted ED value, and the red dashed line represents the significance association threshold. The higher the ED value, the better the association effect of the point.

The comparison rates of the raw pool 1 and pool 2 data with the *C. oleifera* genome sequence were 62.54% and 65.82%, respectively (Table 2). A total of 278,973 variant loci were detected using GATK 4.0, comprising 245,601 SNP and 33,372 indel loci (Figure 4). Using the SNP loci with genotypic differences between pools 1 and 2, we counted the depth of each base in the different bulk groups and calculated the ED value of each locus. The distribution of association values across the chromosomes is shown in Figure 5.

**Figure 6:**
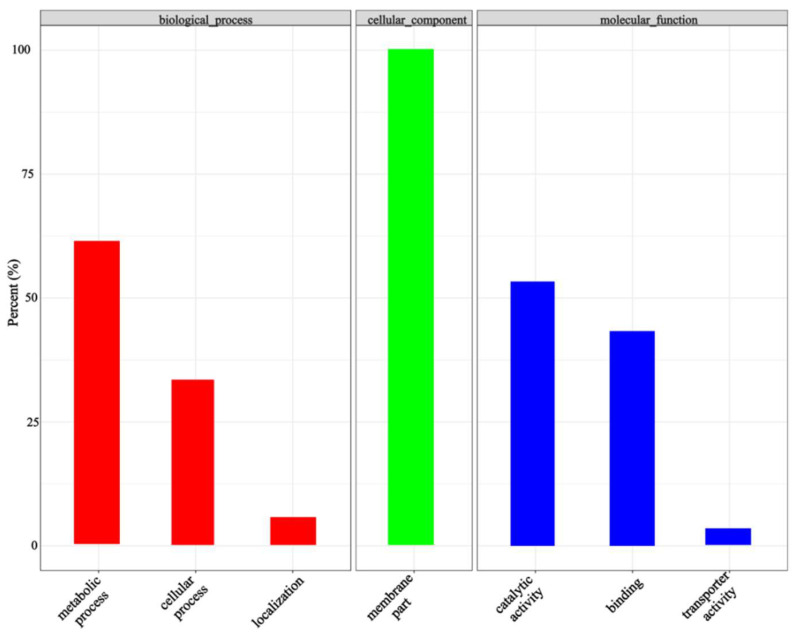
The enriched GO terms in the molecular function, cellular component, and biological process categories.

**Figure 7:**
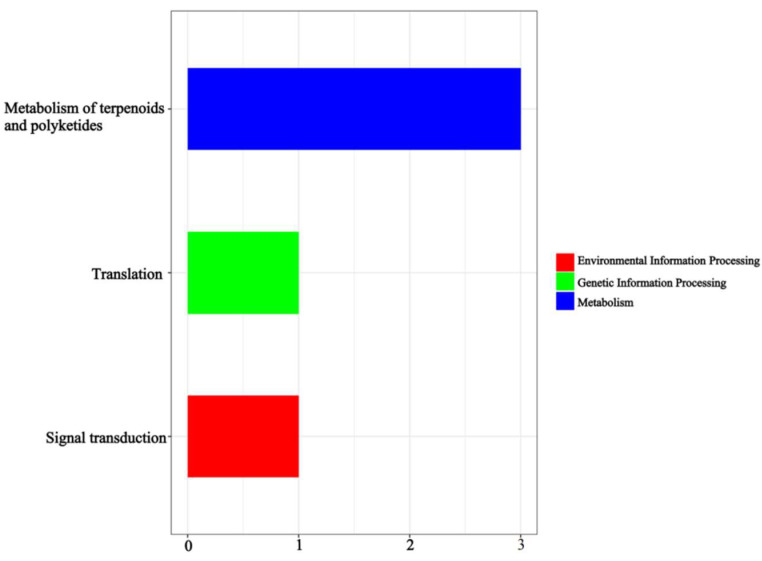
The enriched KEGG terms.

Based on the association threshold, two chromosome association regions were obtained: 140.8–148.4 Mb on chromosome 2 and 35.3–37.3 Mb on chromosome 15 (Table 3). A total of 135 candidate genes from the two chromosome association regions were obtained, and 49 of these genes were predicted to be functionally annotated (based on the Swiss-Prot, Pfam, KEGG, GO, NR, KOG, and TrEMBL databases). Based on a GO enrichment analysis, 25 enriched terms were divided into three ontological categories: molecular function, cellular components, and biological processes (Figure 6). The candidate transcripts for seed ratio may be localized to the membrane, involved in 1,3-β-d-glucan biosynthesis, cellulose biosynthesis, isoprenoid biosynthesis, redox reactions, or transmembrane transport, and have hydrolase (acting on ester bonds), oxidoreductase, cellulose synthase (uridine-diphosphate-forming), and 1,3-β-d-glucan synthase functions. A KEGG pathway analysis was also conducted to determine the possible roles of the DEPs in biological metabolic pathways. The DEPs were significantly enriched in nine KEGG pathways (Figure 7).

Monoterpenoid biosynthesis and tyrosine metabolism were the most enriched pathways. The other seven enriched pathways were mainly related to alpha-linolenic acid metabolism, the peroxisome proliferator-activated receptor signaling pathway, the adenosine 3′,5′-cyclic monophosphate signaling pathway, purine metabolism, phototransduction, salivary secretion, and gastric acid secretion. The product of the DEPs was predicted to contain the LRRNT_2 family, WPP domain, and RRM_1 domain (Figure 7).

### 3.5. Expression of Associated Transcripts/Genes

**Figure 8:**
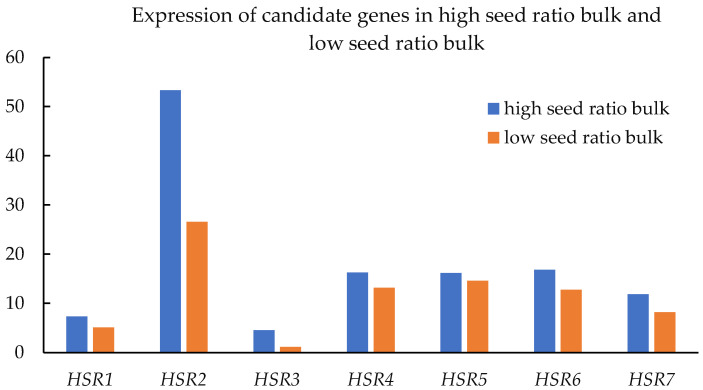
Analysis of the expression of genes associated with high seed ratio. HSR: high seed ratio.

In the association gene analysis, 43 genes were not expressed in either pool, 34 genes were less expressed in pool 1 than in pool 2, and 48 genes were more expressed in pool 1 than in pool 2. Among these genes, 14 were twice as abundant in pool 1 as in pool 2, and 22 were twice as abundant in pool 2 as in pool 1 (Supplement S1). The intersection (blast value less than 0.00001, coverage greater than 40 percent) of the candidate genes resulted in *HSR1*, *HSR2*, *HSR3*, *HSR4*, *HSR5*, *HSR6*, and *HSR7* using the two methods of analysis. The DEGs from two set pairs of cDNA sequencing bulks were enriched with cytochrome P450 CYP76F14 (KOG0156; GO:0055114, *HSR4*, *HSR7*), the gibberellin phytohormone pathway (GO:0016787, *HSR5*), the calcium signaling pathway (GO:0005509, *HSR6*), the polyubiquitin-PPAR signaling pathway (GO:0005515, *HSR2*, *HSR3*), and several main transcription factors (bZIP transcription factor, *HSR1*) in *C. vietnamensis*. The second-generation transcriptome data show that all seven candidate genes were more expressed in the high-seed-ratio bulk than in the low-seed-ratio bulk (Figure 8). The RT-PCR results show that the seven candidate genes were not more highly expressed in the high-seed-ratio plants and were not less expressed in the low-seed-ratio plants. Therefore, for a complex quantitative trait, relevant candidate genes need to be further validated.

## 4. Discussion

Since 2005, this project team has completed phenotypic diversity surveys (flower, fruit and leaf) in 13 plots of *C. vietnamensis*, including Vietnam, Guangdong, Hainan, and Guangxi. The survey data show that the fresh seed ratio of fruits in the seedling population of *C. vietnamensis* was significantly different. Plants with a fresh seed ratio of 24.6–34.3% constitute more than 90%, while those with a fresh seed ratio of >40% represent less than 0.8% (unpublished data). In *C. vietnamensis*, the seed ratio is a complex quantitative trait with a normal distribution, which is significantly associated with four other traits of fruit (seed weight, seed number, fruit weight, and pericarp thickness), and is highly significantly positively correlated with seed weight (*p* < 0.01) and seed number (*p* = 0.012), and significantly negatively correlated with fruit weight and pericarp thickness (*p* < 0.01).

In *C. vietnamensis*, heterozygosity is strong, and genomes are often large and complex. Therefore, studies on the genetic background of *C. vietnamensis* have been limited. For species without reference genomes, RNA-Seq data have been used to obtain genetic information and construct physical maps [40]. In this study, the conjunctive analyses of full-length transcriptome sequencing and BSR-Seq are performed with the F2 separation progeny to study the molecular mechanism of high seed ratio formation in *C. vietnamensis*. Moreover, *C. vietnamensis* and *C. oleifera* var. belong to *Sect. Oleifera Chang Tax*. of *Camellia L* of Theaceae. The alignment results of two bulk reads of *C. vietnamensis* on *C. oleifera* var. genome sequences were 62.54% and 65.82% (Table 2). Based on these results, we used the *C. oleifera* var. genome as a template to screen the *C. vietnamensis* candidate genes for high seed ratio. Two association regions were predicted. One spanned 140.8–148.4 Mb of chromosome 2 and was associated with 97 seed-yield-related candidate genes ranging in length from 278 to 16,628 bp. The other spanned 35.3–37.3 Mb on chromosome 15 and was associated with 38 candidate genes ranging in length from 221 to 16,928 bp.

The DEGs from two set pairs of cDNA sequencing bulks are enriched to cytochrome P450 CYP76F14 (KOG0156; GO:0055114), the gibberellin phytohormone pathway (GO:0016787), the calcium signaling pathway (GO:0005509), the polyubiquitin-PPAR signaling pathway (GO:0005515), and several main transcription factors (bZIP transcription factor) in *C. vietnamensis*.

### 4.1. Cytochrome p450 Regulation in C. vietnamensis Seed Ratio

Cytochrome p450 is currently one of the largest enzyme proteins and is found in a range of plants, from bryophytes to angiosperms, and members of this family play an important role in promoting plant growth and development in a variety of species [41,42]. Several CYP78As have also been found to regulate organ size and development in other plants such as Arabidopsis thaliana [43] and cucurbits [44]. Moreover, OsCYP78A13 regulates grain size by mediating cell-cycle progression to balance the embryo/endosperm size in rice [45]. PaCYP78A9 plays an essential role in the regulation of cherry fruit size [46]. TaCYP72A also had a positive effect on grain number in wheat [47]. In this study, DEGs (HSR4, HSR7) are enriched to cytochrome P450 CYP76F14, KOG0156 and GO:0055114, respectively. The two DEGs may be involved in the formation of camellia seed size, which further affects fruit weight.

### 4.2. Phytohormone Regulation in C. vietnamensis Seed Ratio

GO terms and the KEGG pathway were significantly enriched in *C. vietnamensis*, as revealed by DEG enrichment analysis, including gibberellin receptor (GO:0016787) and plant hormone signal transduction (ko04075, *HSR5*), suggestive of the imperative roles that phytohormones may play in regulating seed ratio development. Auxin and GA have been reported to mediate tomato fruit growth, increasing their contents and signaling accelerated fruit growth [48]. Fruit growth consists of cell division and expansion, with the former being shown to be influenced by auxin signaling. While the regulation of cell expansion is less thoroughly understood, evidence indicates synergistic regulation via both auxin and GAs, with input from additional hormones. In recent years, several genes in the gibberellin metabolism and signaling pathway have been shown to play an essential role in regulating fruit growth/size in Arabidopsis [49]. In this study, *HSR5* exhibited substantially higher gene expression in the high seed ratio bulk than in low seed ratio bulk, suggestive of their positive regulatory roles in seed ratio development in *C. vietnamensis*.

### 4.3. Ubiquitin-Specific Protease Regulation in C. vietnamensis Seed Ratio

Ubiquitin-specific protease (UBP) is a highly conserved protein family in eukaryotes, which plays an important role in protein de-ubiquitination [50]. Several factors involved in ubiquitin-related activities have been known to determine seed size in Arabidopsis and rice. The knockout mutants of UBC22 in Arabidopsis had reduced silique length and seed number per silique [51]. Thus, the polyubiquitin-PPAR signaling pathway (GO:0005515, *HSR2*) may be involved in the formation of the seed ratio.

## 5. Conclusions

In this study, we characterized C. vietnamensis plants with high and low seed ratio traits. To screen for candidate genes and determine the molecular mechanisms underlying high seed ratio, BSR and full-length transcriptome sequencing were performed. The results show that the trait is controlled by multiple genes. Two candidate regions related to high seed ratio were predicted. We found that cytochrome P450, gibberellin, the calcium signaling pathway, the polyubiquitin-PPAR signaling pathway, and the bZIP transcription factor play an important role in affecting high seed ratio in C. vietnamensis. Seven genes were promising candidates responsible for the high-seed-ratio plants. However, the functional validation of these genes requires investigation in future study.

## Figures and Tables

**Table 1:** Alignment results of two bulk reads on full-length transcripts.

Sample	Total Reads (bp)	Pair Reads	Reference Sequence	Aligned Concordantly 0 Times ^a^	Aligned Concordantly Exactly 1 Time ^b^	Aligned Concordantly > 1 Times ^c^	Total Alignment Ratio ^d^
pool 1	31,123,912	31,123,912 (100.00%)	full-length transcript	15,282,712 (49.10%)	6,617,832 (21.26%)	9,223,368 (29.63%)	60.41%
pool 2	37,549,983	37,549,983 (100.00%)		18,946,996 (50.46%)	8,326,225 (22.17%)	10,276,762 (27.37%)	62.06%

Note: pair reads: in paired-end sequencing, the number and percentage of read pairs that can be paired. ^a^ The number and percentage of unmatched read pairs. ^b^ The number and percentage of uniquely aligned read pairs (both ends are aligned). ^c^ The number and percentage of read pairs in multiple alignments (both ends are aligned). ^d^ Overall data comparison rate.

**Table 2:** Alignment results of two bulks Reads on whole genome sequences of *C. oleifera* var.

Sample	Total Reads (bp)	Pair Reads		Aligned Concordantly 0 Times ^a^	Aligned Concordantly Exactly 1 Time ^b^	Aligned Concordantly > 1 Times ^c^	Total Alignment Ratio ^d^
pool 1 (High)	31,123,912	31,123,912 (100.00%)	*Camellia oleifera* (eudicots) genome	18,080,834 (58.09%)	5,743,157 (18.45%)	7,299,921 (23.45%)	62.54%
pool 2 (Low)	37,549,983	37,549,983 (100.00%)		22,110,840 (58.88%)	6,036,660 (16.08%)	9,402,483 (25.04%)	65.82%

Note: pair reads: in paired-end sequencing, the number and percentage of read pairs that can be paired. ^a^ The number and percentage of unmatched read pairs. ^b^ The number and percentage of uniquely aligned read pairs (both ends are aligned). ^c^ The number and percentage of read pairs in multiple alignments (both ends are aligned). ^d^ Overall data comparison rate.

**Table 3:** Linkage intervals associated with candidate genes of seed ratio.

Chromosome	Start (Mb)	End (Mb)	Length (Mb)	Candidate Gene Number	SNP Number	InDel Number
Chr2	140.8	148.4	7.6	97	865	42
Chr15	35.3	37.3	2.0	38	24	2

## Data Availability

Sequence Read Archive (SRA) submission: SUB11950986, BioProject Number: RNA-seq for bulked segregant analysis (PRJNA881645).

## Supplementary Materials

The following supporting information can be downloaded at: https://www.mdpi.com/article/10.3390/cimb45010022/s1, Figure S1: Prediction results of lncRNA of the full-length transcript of *C. vietnamensis*; Figure S2: GO annotation classification map of the full-length transcriptome sequence; Figure S3: KEEG annotation classification map of the full-length transcriptome sequence; Table S1: Statistical results for non-redundant high quality full-length transcripts.
