# Supplementary material for: BSR and Full-Length Transcriptome Approaches Identified Candidate Genes for High Seed Ratio in Camellia vietnamensis
Source: Curr Issues Mol Biol. 2022 Dec 31;45(1):311–26. doi: 10.3390/cimb45010022 (PMC9857833; doi:10.3390/cimb45010022)
Supplement: Supplementary file 1 [file cimb-45-00022-s001.zip › cimb-2091666-sup.pdf]

Supplementary Material

1 Supplementary Figures and Tables

1.1 Supplementary Figures

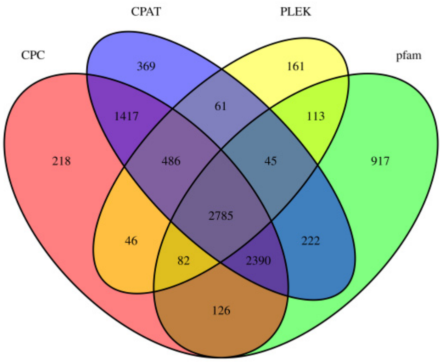

Supplementary Figure S1. Prediction results of lncRNA of the full-length transcript of *C. vietnamensis*

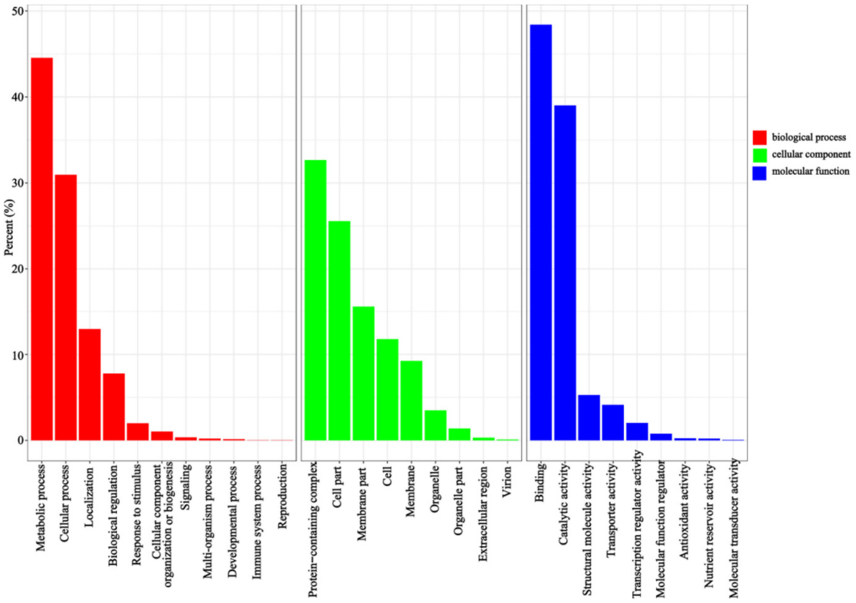

Supplementary Figure S2. GO annotation classification map of the full-length transcriptome sequence

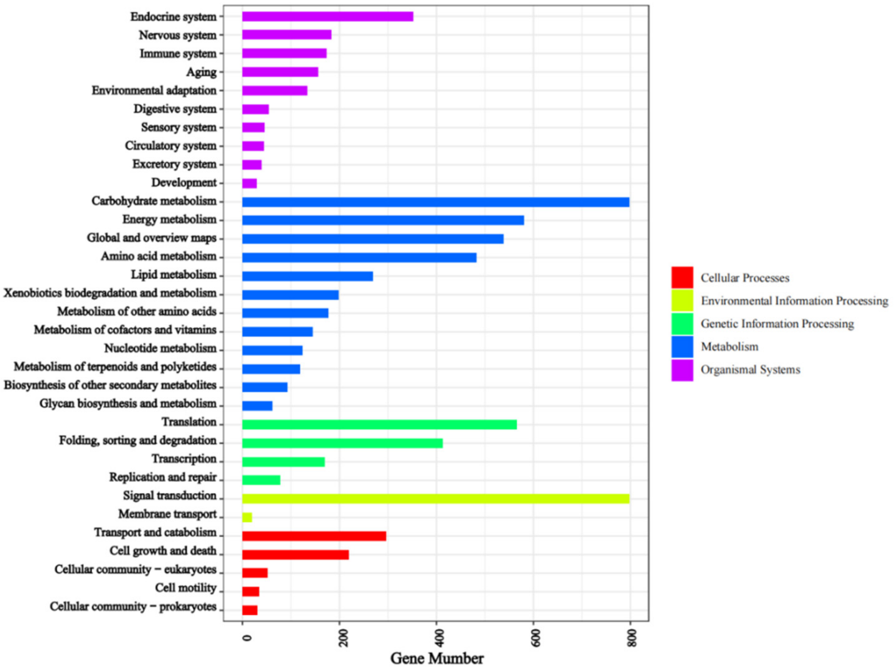

Supplementary Figure S3. KEEG annotation classification map of the full-length transcriptome sequence

2.2 Supplementary Tables

Supplementary Table S1. Statistical results for non-redundant high quality full-length transcripts

Seq_ number	Total Length（bp）	<1kb	>1kb&<2kb	>2kb&3kb	>3kb	N50	N90	Mean	Median	Max	Min
14152	12403692	9835	3935	350	32	997	515	876	770	4719	107
